# Persulfide Biosynthesis Conserved Evolutionarily in All Organisms

**DOI:** 10.1089/ars.2023.0405

**Published:** 2023-11-13

**Authors:** Seiryo Ogata, Tetsuro Matsunaga, Minkyung Jung, Uladzimir Barayeu, Masanobu Morita, Takaaki Akaike

**Affiliations:** Department of Environmental Medicine and Molecular Toxicology, Tohoku University Graduate School of Medicine, Sendai, Japan.

**Keywords:** persulfide, polysulfide, supersulfide, CARS, CPERS, evolution

## Abstract

**Significance:**

Persulfides/polysulfides are sulfur-catenated molecular species (*i.e.*, R-S_n_-R′, *n* > 2; R-S_n_-H, *n* > 1, with R = cysteine, glutathione, and proteins), such as cysteine persulfide (CysSSH). These species are abundantly formed as endogenous metabolites in mammalian and human cells and tissues. However, the persulfide synthesis mechanism has yet to be thoroughly discussed.

**Recent Advances:**

We used β-(4-hydroxyphenyl)ethyl iodoacetamide and mass spectrometry to develop sulfur metabolomics, a highly precise, quantitative analytical method for sulfur metabolites.

**Critical Issues:**

With this method, we detected appreciable amounts of different persulfide species in biological specimens from various organisms, from the domains Bacteria, Archaea, and Eukarya. By using our rigorously quantitative approach, we identified cysteinyl-tRNA synthetase (CARS) as a novel persulfide synthase, and we found that the CysSSH synthase activity of CARS is highly conserved from the domains Bacteria to Eukarya. Because persulfide synthesis is found not only with CARS but also with other sulfotransferase enzymes in many organisms, persulfides/polysulfides are expected to contribute as fundamental elements to substantially diverse biological phenomena. In fact, persulfide generation in higher organisms—that is, plants and animals—demonstrated various physiological functions that are mediated by redox signaling, such as regulation of energy metabolism, infection, inflammation, and cell death, including ferroptosis.

**Future Directions:**

Investigating CARS-dependent persulfide production may clarify various pathways of redox signaling in physiological and pathophysiological conditions and may thereby promote the development of preventive and therapeutic measures for oxidative stress as well as different inflammatory, metabolic, and neurodegenerative diseases. *Antioxid. Redox Signal.* 39, 983–999.

## Introduction

Persulfides and polysulfides, which we found in abundance, are polymeric sulfur-containing metabolites with sulfur catenation that occur in biological organisms; these compounds include, for example, R-S_n_-R′, *n* > 2, and R-S_n_-H, *n* > 1, with R = cysteine, glutathione, or proteins (Akaike et al., 2017). They are produced ubiquitously not only in primitive unicellular organisms and prokaryotes but also in eukaryotes and higher organisms such as plants and mammals, including humans (Akaike et al., 2017; Fukuto et al., 2018; Ida et al., 2014).

Persulfides/polysulfides that are typically and widely found in various organisms include cysteine hydropersulfide/polysulfide (CysS-S_n_-H), glutathione hydropersulfide/polysulfide (GS-S_n_-H), and glutathione trisulfide/polysulfide, which are more redox-active than are other thiols and disulfides (Akaike et al., 2017; Fukuto et al., 2018; Fukuto et al., 2012; Ida et al., 2014; Peng et al., 2017; Shimizu et al., 2017).

For example, persulfides act as potent antioxidants and redox-signaling molecules (Akaike et al., 2017; Fukuto et al., 2018; Ida et al., 2014). Persulfides also reduce stress-induced cellular senescence and improve chronic heart failure (Nishida et al., 2012). Cysteine persulfide (CysSSH) behaves as a strong nucleophile and antioxidant and plays an essential role in regulating cellular redox balance and redox signaling (de Beus et al., 2004; Gao et al., 2015; Ida et al., 2014; Kasamatsu et al., 2016; Mustafa et al., 2009; Vandiver et al., 2013; Yang et al., 2015). Several papers have also reported that various proteins and enzymes at their specific cysteine residues possess cysteine polysulfides, including CysSSH, that is, protein polysulfidation.

Aside from the redox signaling regulatory mechanism, another possible function of protein polysulfidation is protecting protein thiol residues against irreversible chemical modification that is caused by oxidants and electrophiles. This article will provide an overview of the most recent updates pertaining to the unique mechanism of persulfide biosynthesis and physiological roles of persulfides/polysulfides generated by the domains Bacteria, Archaea, and Eukarya.

## Polymeric Sulfurs Supporting Ancient Life During Evolution

**FIG. 1.**
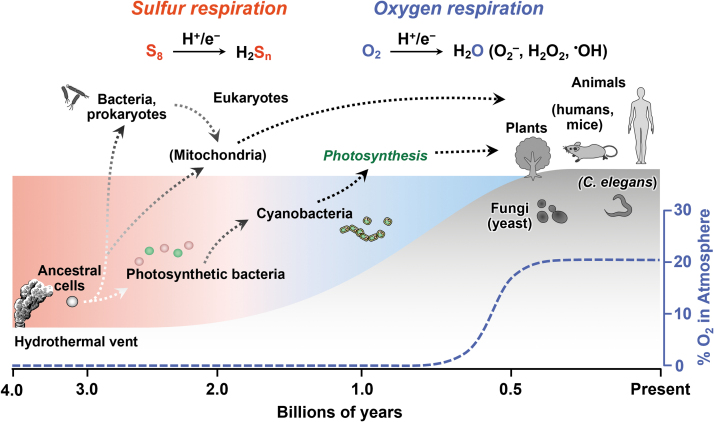
**Evolutionary conservation of sulfur respiration in all organisms.** Ancestral cells or organisms that initially appeared in the hydrothermal vent underwent sulfur respiration. Even ancient photosynthetic bacteria likely generated polysulfides such as S_8_, which evolved and kept up with energy metabolic functions in the mitochondria of higher organisms during evolution. Transformation of the original respiration into a novel type of sulfur respiration occurred, which utilized sulfur-containing amino acid cysteine persulfides rather than inorganic S_8_ as an electron acceptor. Our interpretation here is that an electron is transferred from sulfur to oxygen, so we call this process an S-O mixed-type hybrid respiration. This illustration presents the comprehensive history of sulfur respiration in the various organisms that live on earth. *C. elegans*, *Caenorhabditis elegans*.

**FIG. 2.**
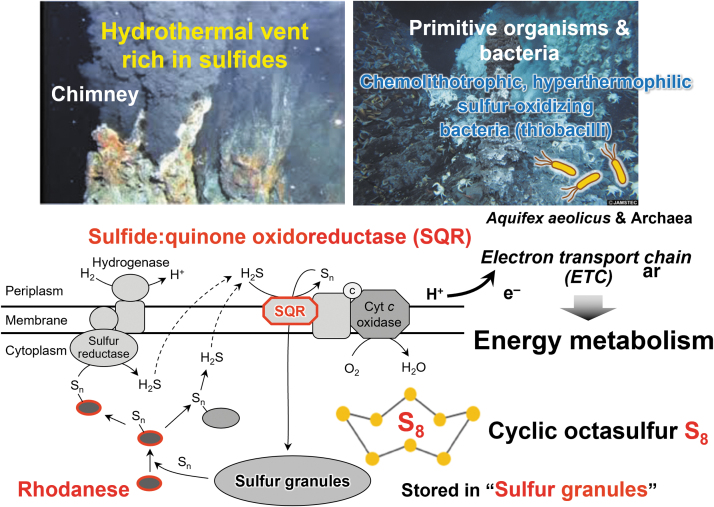
**H_2_S in the hydrothermal vent: A fundamental component of energy metabolism of primitive organisms.** One well-known major type of fundamental energy metabolism in organisms is mediated by sulfur, which is typically used by primitive organisms such as bacteria that live in the hydrothermal vent. These microorganisms, called chemolithotrophic bacteria, are also known as sulfur/sulfide-oxidizing bacteria—*Thiobacillus*. These bacteria can utilize H_2_S as an electron donor to produce a proton gradient and energy *via* an ETC, which leads to sulfur allotropes such as S_8_ and ATP production. ETC, electron transport chain; H_2_S, hydrogen sulfide (Arnulf, 2006). Photographs taken from the Japan Agency for Marine-Earth Science and Technology (JAMSTEC), https://www.jamstec.go.jp/e/ [accessed May 30, 2023].

About 4 billion years ago, primitive cells that gave rise to life appeared on earth under anoxic conditions. Even in the absence of oxygen, the element sulfur and its allotropes serve as electron acceptors that are required for energy production in many aerobic organisms (Figs. 1 and 2) (Olson, 2020). Throughout the long history of life on earth, sulfur-dependent energy metabolism has continuously contributed to the evolution of both anaerobic and aerobic organisms.

Previous studies of sulfur metabolism have focused mainly on the biosynthesis and metabolism of sulfur-containing amino acids and proteins, in terms of derivatives of thiol residue oxidation, especially under aerobic and oxidative stress conditions. In an emerging field of sulfur metabolism; however, several researchers began to investigate the physiological role of hydrogen sulfide (H_2_S), as previously discussed (Mishanina et al., 2015; Zivanovic et al., 2020).

H_2_S was reportedly merely a degraded product of diverse physiological polymeric sulfurs. However, the exact mechanisms of the biosynthesis and regulation of H_2_S are still unknown. In addition, an important question remains: how various organisms might utilize persulfides/polysulfides for biological/physiological functions (Akaike et al., 2017; Fukuto et al., 2018; Ida et al., 2014).

By developing a new quantitative analytical system for reactive sulfur metabolites, we successfully detected high amounts of persulfides/polysulfides in biological milieus derived from various species (Akaike et al., 2017; Ida et al., 2014). We reported that the persulfide levels in the cells are in a sub-millimolar range (Matsunaga et al., 2023). In fact, we demonstrated that cysteinyl-tRNA synthetase (CARS), an aminoacyl-tRNA synthetase, functions as a novel CysSSH-producing enzyme (Akaike et al., 2017).

Our biochemical analysis indicated that CARS could synthesize CysSSH from its substrate cysteine in *Escherichia coli*, mice, and humans. In addition, we found that CysSSH synthase activity was conserved in the domains Bacteria, Archaea, and Eukarya. These findings indicate that CysSSH biosynthesis pathways are well conserved evolutionarily in all organisms.

## Rigorous Quantitative Analysis of Persulfides/Polysulfides Formed in Organisms

CysSSH has unique reactive properties that differ from those of cysteine. The persulfide/polysulfide derivatives of cysteine exhibit both nucleophilic and electrophilic properties (Abdolrasulnia and Wood, 1980; Fletcher and Robson, 1963; Parker and Kharasch, 1959). On the basis of the potential nucleophilicity of persulfides/polysulfides as well as the nucleophilicity of thiol residues, we can determine precisely the various persulfide/polysulfide species in the cells.

**FIG. 3.**
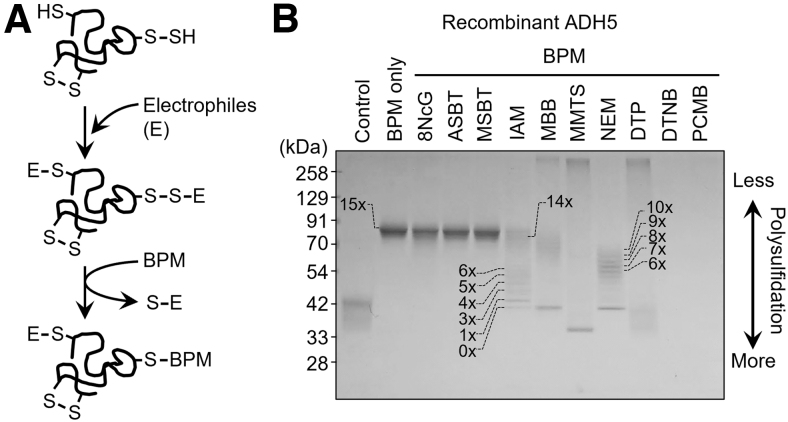
**Detection of protein persulfidation by using the PMSA. (A)** Schematic drawing showing the reaction mechanism in the PMSA. In the initial step of the reaction, both thiol (-SH) and polysulfide (-S_n_-H) groups of the cysteine residues in a protein are alkylated by electrophiles (E). In the second reaction step, the PEGylation probe BPM selectively attacks alkylated polysulfides at proximal sulfur atoms. Polysulfide-rich proteins acquire PEGylation and demonstrate low gel mobility. **(B)** Detection of protein persulfidation in the WT ADH5 by using the original PMSA. Numbers on the gels and blots indicate expected numbers of PEG moieties in the protein, which were inferred from the mobility shift distance. Proteins were detected by means of CBB staining. 8NcG, 8-nitro-cGMP; ADH5, alcohol dehydrogenase 5; ASBT, 2-aminosulfonyl benzothiazole; BPM, biotin-PEG-MAL; CBB, Coomassie Brilliant Blue; DTNB, 5,5′-dithiobis(2-nitrobenzoic acid); DTP, 4,4′-dithiopyridine; IAM, iodoacetamide; MBB, monobromobimane; MMTS, methyl methanethiosulfonate; MSBT, 2-methylsulfonyl benzothiazole, NEM, *N*-ethylmaleimide; PCMB, *p*-chloromercuribenzoic acid; PEG, polyethylene glycol; PMSA, PEG-conjugated maleimide labeled gel shift assay; WT, wild type.

In fact, we developed a valuable method for accurately detecting persulfides and polysulfides formed in proteins that was based on a biotin-polyethylene glycol-maleimide (biotin-PEG-MAL)-labeled gel shift assay (PMSA) (Jung et al., 2016). With this PMSA, we reported that many recombinant proteins such as alcohol dehydrogenase 5 (ADH5) and endogenous proteins expressed by various types of cells in culture are polysulfidated (Fig. 3) (Akaike et al., 2017).

Accumulating evidence, therefore, suggests that biologically formed organic (dialkyl) polysulfides (R-S_n_-R′) freely undergo alkaline hydrolysis, which results in the formation of both nucleophilic hydropolysulfide (R-S_n_-H) species and electrophilic sulfenic acid (R′-SOH) species. Polysulfides maintain a hydrolysis-induced steady-state equilibrium even in aqueous physiological environments.

This particular property means that polysulfide chemistry and biology are more complex than previously thought. The hydrolysis equilibrium of polysulfides, for example, shifts to the right in the presence of electrophiles. Strong electrophilic alkylating agents, for example, *N*-ethylmaleimide and monobromobimane, enhance polysulfide hydrolysis considerably, which results in greater polysulfide degradation and artifactual formation of *bis-S*-alkylated adducts in the absence of free H_2_S.

Our earlier study found that tyrosine and the hydroxyphenyl-containing derivative β-(4-hydroxyphenyl)ethyl iodoacetamide (HPE-IAM) had stabilizing effects on various polysulfide residues formed in CysSSH-related low-molecular-weight (LMW) species, such as glutathione polysulfides (Akaike et al., 2017; Numakura et al., 2017). The protection against hydrolysis was probably caused by effects of the hydroxyphenyl moiety of HPE-IAM on alkaline hydrolysis of polysulfides.

**FIG. 4.**
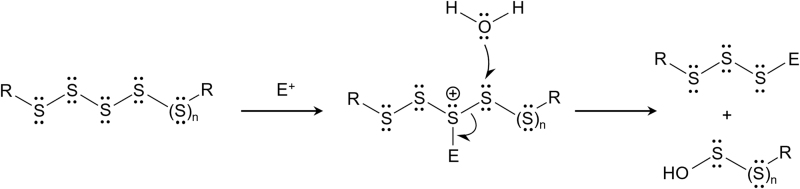
**Polysulfide hydrolysis promoted by electrophiles (E).** Formation of an electrophile–sulfur adduct may initiate the heterolytic cleavage of the S-S bond mediated *via* the nucleophilic attack by water.

This hydrolysis occurred by means of heterolytic scission that was triggered by the action of hydroxyl anions on polysulfides that are split into thiolates and sulfenic acids, with alkylating reagents (*e.g.,* IAM) and dimedone enhancing the hydrolysis (Fig. 4). In addition, simple amino acid tyrosine prevented electrophilic degradation in alkaline condition (Hamid et al., 2019).

It is also interesting to note that polysulfides on hydrolysis yield sulfenic acids that can be scavenged by cellular thiols. Alternatively, the direct reaction between the polysulfides and thiols yields hydropersulfides and H_2_S as well. These reactions have been discussed elsewhere in earlier reports (Brown and Bowden, 2022; Switzer et al., 2021; Wu et al., 2023).

For example, polysulfides could induce thiol oxidation, which, in turn, might promote the cellular oxidative stress. In fact, polysulfides-driven thiol oxidation was demonstrated for cellular oxidative stress sensors: peroxiredoxin 2 and protein tyrosine phosphatase 1B (Dóka et al., 2020) and phosphatase and tensin homologue deleted on chromosome 10 (Greiner et al., 2013).

**FIG. 5.**
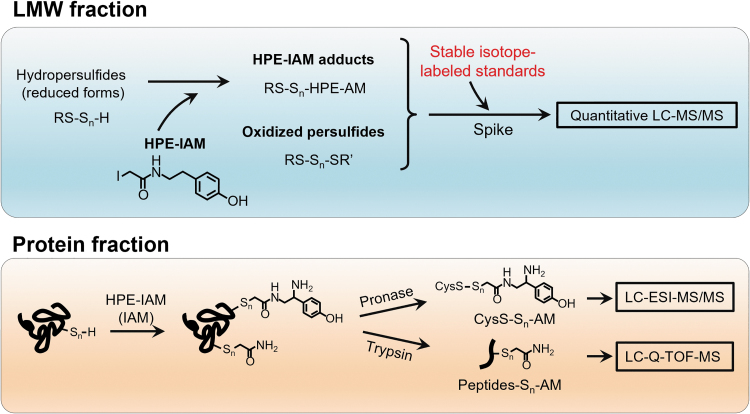
**Sulfur metabolome and proteome analysis developed with HPE-IAM and LC-ESI-MS/MS.** Schematic representation of the quantitative detection of LMW persulfides/polysulfides by using HPE-IAM as a trapping agent and LC-ESI-MS/MS analysis (*upper panel*) and quantitative detection of protein persulfides/polysulfides by means of trypsin digestion and LC-Q-TOF-MS (*lower panel*). ESI, electrospray ionization; HPE-IAM, β-(4-hydroxyphenyl)ethyl iodoacetamide; LC, liquid chromatography; LMW, low-molecular-weight; MS/MS, tandem mass spectrometry; Q-TOF, quadrupole time-of-flight.

For quantitative detection of LMW persulfides/polysulfides and protein persulfides/polysulfides, we developed a method that utilized HPE-IAM as a trapping agent and liquid chromatography (LC)-electrospray ionization (ESI)-tandem mass spectrometry (MS/MS) analysis (Fig. 5) (Akaike et al., 2017; Numakura et al., 2017).

By using different internal standards that were labeled with stable isotopes, we developed this quantitative LC-ESI-MS/MS analysis, which allowed us not only to quantify LMW persulfides/polysulfides but also to identify the polysulfidation of various proteins, such as glyceraldehyde-3-phosphate dehydrogenase (GAPDH) and ADH5, that are highly polysulfidated in cells.

In addition, LC-ESI-MS analysis of HPE-IAM-labeled ADH5 after pronase digestion revealed that 70% of the cysteine residues in ADH5 were endogenously polysulfidated. Also, LC-quadrupole time-of-flight MS analysis identified the polysulfidation site as well as the sulfur chain length of the proteins (Fig. 5). Certain studies suggested that protein polysulfidation may be involved in the regulation and maintenance of the activities of several enzymes (Jarosz et al., 2015; Jung et al., 2016; Millikin et al., 2016).

Thus, establishing an analytical method for persulfides/polysulfides led to significant progress in the quantification of polymeric sulfurs in cells and tissues and will promote better understanding of the biosynthesis and physiological functions of these polymeric sulfurs (Akaike et al., 2017).

## Co-Translational Persulfide Production and Protein Polysulfidation in Organisms

As described earlier, our PMSA and LC-MS/MS analyses clearly indicated that different kinds of proteins are highly polysulfidated endogenously (Akaike et al., 2017; Dóka et al., 2016; Ida et al., 2014; Jung et al., 2016; Ono et al., 2014). To clarify how protein polysulfidation occurs in cells, we studied whether CARS can catalyze the direct incorporation of CysSSH or CysS-S_n_-H into tRNA. CARS is an enzyme that produces cysteinyl-tRNA (Cys-tRNA) *via* cysteine and aminoacyl-tRNA (Carter, 1993; Guo et al., 2010; Woese et al., 2000).

Our work indicated, for the first time, that CARS from *E. coli* (EcCARS) produced CysSSH directly from cysteine as a substrate as well as CysSSH-tRNA by using the resultant CysSSH as a substrate. CysSSH-tRNA can be a substrate for ribosomal protein synthesis as CysS-tRNA and can result in translation-coupled protein polysulfidation of newly synthesized proteins.

This translation-coupled co-translational protein polysulfidation was clearly demonstrated by puromycin-associated nascent chain proteomics (PUNCH-P), as recently reported (Aviner et al., 2014), with PUNCH-P modified for polysulfide proteomics (PUNCH-PsP) (Akaike et al., 2017).

By using this PUNCH-PsP approach, we identified intact forms of CysS-S_n_-H residues in nascent peptides of GAPDH occurring only in *E. coli* ribosomes. In fact, the PUNCH-PsP assay showed significant polysulfidation at the Cys^247^ residue of the nascent peptides of GAPDH protein just after synthesis in *E. coli* ribosomes. We successfully recovered CysSSH and CysSSH residues for all cysteine residues in all native proteins, for example, with the native whole GAPDH protein.

Polysulfidation affected more than 60% of the Cys^247^ residues of the mature protein. Therefore, we now know that widespread cysteine polysulfidation occurs co-translationally in ribosomes and is maintained physiologically in mature proteins in cells.

## Persulfide Biosynthesis Pathways in Prokaryotes

CARS is involved in the translation process as it catalyzes the synthesis of Cys-tRNA, that is, protein translation. By means of our rigorous and extensive investigation of protein polysulfidation, we discovered that CARS has CysSSH synthase (CPERS) activity and is solely involved in LMW biosynthesis of persulfides/polysulfides and protein polysulfidation (Akaike et al., 2017).

Some aminoacyl-tRNA synthetases reportedly have physiological functions other than translation, that is, a moonlighting function (Guo et al., 2010; Wakasugi and Schimmel, 1999). For example, human tyrosyl-tRNA synthetase can be divided into two fragments with specific cytokine activities. The endothelial monocyte-activating polypeptide II-like carboxy-terminal domain has strong leukocyte and monocyte chemotactic activities and stimulates the production of myeloperoxidase, tumor necrosis factor-α, and tissue factor.

The catalytic amino-terminal domain binds to the interleukin-8 type A receptor and serves as an interleukin-8-like cytokine. In cell culture, under apoptotic conditions, secretion of the full-length enzyme occurs, and leukocyte elastase, an extracellular protease, can generate the two cytokine activities. tRNA synthetase secretion may contribute to apoptosis by both stopping translation and producing the needed cytokines (Wakasugi and Schimmel, 1999).

**FIG. 6.**
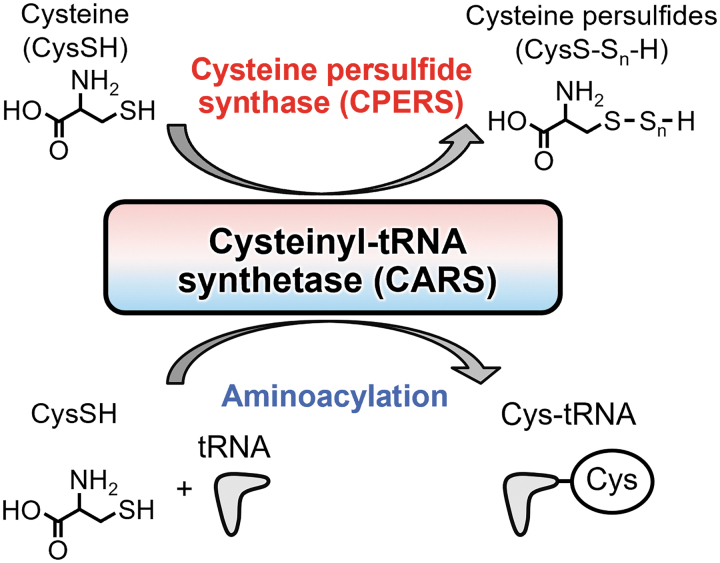
**Sulfur metabolic pathway coupled to translation by the CysSSH-producing enzyme CARS.** CARSs produce CysSSH from cysteine. CARSs can convert cysteine to CysSSH *via* a PLP-dependent process using a second cysteine as the sulfur atom donor (independent of ATP and tRNA). The CARS-synthesized CysSSH can then form a tRNA-bound CysSSH adduct (also *via* CARS catalysis), which would result in the incorporation of CysSSH into proteins, thereby generating a protein containing a hydropersulfide function. The CysSSH-producing activity of CARSs is critically involved in translation-coupled protein persulfidation. CARS, cysteinyl-tRNA synthetase; CysSH, cysteine; CysSSH, cysteine persulfide; CysS-S_n_-H, cysteine hydropersulfide/polysulfide; Cys-tRNA, cysteinyl-tRNA; PLP, pyridoxal phosphate.

Thus, the CPERS activity of CARS is a relatively new function of aminoacyltransferases other than translation. Indeed, CARS efficiently produces CysSSH by using cysteine as a substrate in a mechanism that is independent of the generally known aminoacyltransferase reaction (Fig. 6).

The production of CysSSH by EcCARS depended on the addition of pyridoxal phosphate (PLP). Also, not only CysSSH but also CysSSSH are found to be produced by EcCARS; although it is still unclear whether the CysSSSH is catalytically formed by EcCARS or chemically generated through a bimolecular reaction of CysSSH that is the primary catalytic product of EcCARS (Akaike et al., 2017).

**FIG. 7.**
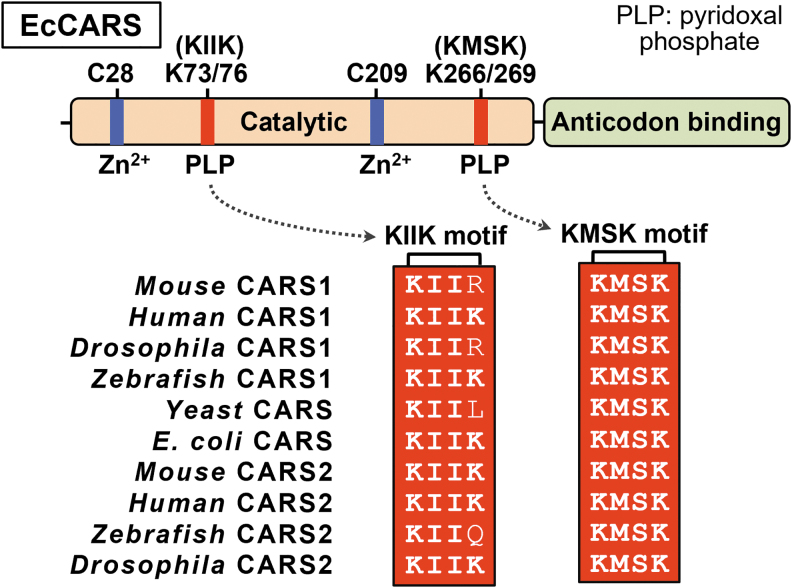
**Domain structure and key amino acid residues involved in the aminoacylation and PLP binding of CARS.** General structure (*upper panel*) and conserved amino acid alignments (*lower panel*) of CARS from bacteria to humans. CARS1, cytosolic cysteinyl-tRNA synthetase; CARS2, mitochondrial cysteinyl-tRNA synthetase.

Proteomic analysis indicated the existence of several preferential sites for PLP binding in EcCARS, including lysine (Lys) residues in the ^73^KIIK^76^ and ^266^KMSK^269^ motifs (Akaike et al., 2017). Sequence data also suggested that several Lys residues in the KIIK and KMSK motifs were highly conserved in EcCARS and homologs in different organisms, including mammals (Akaike et al., 2017) (Fig. 7). Two well-conserved cysteine residues were at the active center during tRNA aminoacylation and were bound to Zn^2+^ (Zhang et al., 2003).

Several Lys mutants of this enzyme were generated to clarify the role of PLP bound to EcCARS, and CysS-S_n_-H formation and translation were measured. As a result, synthesis of CysSSH and CysSSSH was significantly reduced in the mutants K73A, K76A, K266A, and K269A, and the double mutants K73/76A and K266/269A, in which EcCARS Lys was converted to alanine, compared with the wild type (WT). All these mutant enzymes were evaluated by using the PUREfrex cell-free protein synthesis assay (Akaike et al., 2017).

Their protein synthesis potential was unchanged. The amount of PLP bound to EcCARS was quantified by means of LC-ESI-MS/MS using a 2,4-dinitrophenylhydrazine-based method. This LC-ESI-MS/MS analysis showed that the amount of PLP bound to the enzyme was reduced in Lys mutants compared with WT and, more important, the amount of PLP correlated well with persulfide production activity.

In contrast, cysteine to serine or cysteine to aspartate mutants, such as C28S, C28D, C209S, and C209D and the double mutants C28/209S and C28/209D, showed no change in persulfide production activity, even though protein synthesis and translation activities were greatly reduced (Akaike et al., 2017).

Computational modeling of the three-dimensional structure of EcCARS confirmed the binding of PLP to specific Lys residues in the ^73^KIIK^76^ and ^266^KMSK^269^ motifs of EcCARS (Akaike et al., 2017). This modeling also showed that the motif for PLP binding was in a position different from that of the ATP-binding HIGH motif and the Zn^2+^-binding active site for Cys-tRNA^Cys^ biosynthesis.

Because the Lys mutations in the KIIK and KMSK motifs significantly affected the persulfide synthetic activity of EcCARS, mutations in any of the four Lys residues likely cause a commensurate change in PLP binding ability and stability. These data suggest that EcCARS is a unique and efficient CPERS enzyme with an independent catalytic function in aminoacyl-tRNA biosynthesis.

Only 30 years ago, the domain Archaea was established as the third domain of life after Bacteria and Eukarya (Woese and Fox, 1977; Woese et al., 1990). One interesting characteristic of archaea organisms is that they lack CARS. In most organisms—in the Bacteria and Eukarya—cysteine biosynthesis and Cys-tRNA^Cys^ formation are performed separately by cysteine synthase and CARS, respectively. However, methanogens utilize a tRNA^Cys^-dependent cysteine biosynthetic pathway (Sauerwald et al., 2005).

**FIG. 8.**
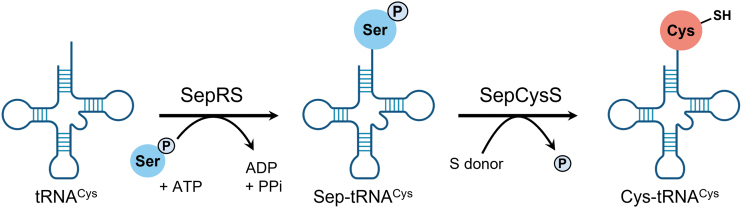
**Scheme of Cys-tRNA^Cys^ formation in archaea.** Sep, *O*-phosphoserine; SepCysS, Sep-tRNA:Cys-tRNA synthase; SepRS, phosphoseryl-tRNA synthetase.

In these archaea organisms, tRNA^Cys^ is first misacylated to *O*-phosphoserine (Sep)-tRNA by phosphoseryl-tRNA synthetase (SepRS), and then Sep-tRNA^Cys^ is converted to Cys-tRNA^Cys^ by Sep-tRNA:Cys-tRNA synthase (SepCysS), a two-step process (Liu et al., 2016; Liu et al., 2014; Rauch and Perona, 2016; Sauerwald et al., 2005) (Fig. 8).

Archaea organisms lack CARS, but many such archaea organisms utilize sulfur compounds and contain persulfides as electron donors or acceptors for energy production (Arnulf, 2006). Therefore, although the mechanisms of persulfide production in Archaea are unknown, SepCysS and other aminoacyl-tRNA synthetases may be involved in persulfide production.

## Persulfide Biosynthesis Pathways in Eukaryotes

Eukaryotes typically utilize two distinct isoforms of aminoacyl-tRNA synthetase, one for cytosolic protein synthesis and the other one for mitochondrial protein synthesis. However, the genome of the budding yeast *Saccharomyces cerevisiae* has only one CARS gene (YNL247W, or *CRS1*). An earlier study reported that *CRS1* encodes both isoforms, cytosolic and mitochondrial.

Two analytical methods, the 5′ complementary DNA end method and the green fluorescent protein reporter gene method, showed that expression of the yeast *CRS1* gene yields two classes of mRNAs *via* alternative transcription starts: a long mRNA possessing a mitochondrial targeting sequence and a short mRNA without this targeting sequence.

In our study, mitochondrial Crs1 was the translation product from the first initiation AUG codon on the long mRNA, and cytosolic Crs1 was from the second in-frame AUG codon on the short mRNA. Genetic analysis and a chromatin immunoprecipitation assay showed that the transcription factor heme activator protein (Hap) complex, which is associated with mitochondrial biogenesis, governed the transcription start sites of the *CRS1* gene.

In addition, Hap complex-dependent initiation was regulated according to the requirements of mitochondrial energy production. Our results, thus, showed energy-dependent initiation of alternative transcription of *CRS1* that produced two Crs1 isoforms, a finding that suggests the possible involvement of Crs1 in mitochondrial energy metabolism in yeast (Nishimura et al., 2019).

**FIG. 9.**
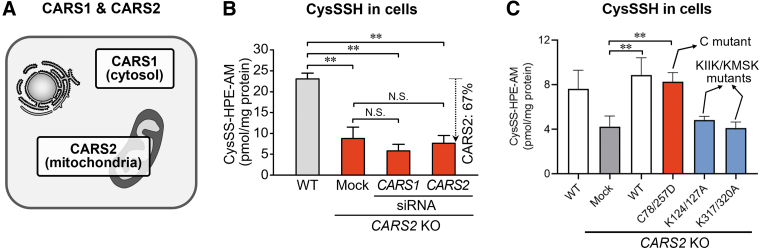
**Endogenous formation of persulfides in HEK293T cells. (A)** Two different CARSs exist in mammals: CARS1 (cytosolic) and CARS2 (mitochondrial). **(B)** Intracellular levels of CysSSH in WT and *CARS2* KO cells with *CARS1* or *CARS2* knocked down. Data are means ± SD (*n* = 3). **p < 0.01; N.S., not significant. **(C)** Production of CysSSH in *CARS2* KO cells with WT or CARS2 C and K mutants added back. The data are means ± SD (*n* = 3). **p < 0.01 *versus CARS2* KO mock. KO, knockout; SD, standard deviation; siRNA, small interfering RNA.

In contrast, two types of mammalian CARS generally exist: CARS1 (cytosolic) and CARS2 (mitochondrial) (Fig. 9A) (Coughlin et al., 2015; Hallmann et al., 2014). Both CARS (we tested mouse CARS1 and human CARS2) had strong CysSSH-producing activity that depended on PLP. Also, a high correlation existed between CPERS activity and PLP content of CARS2 after the treatment of CARS2 with various amounts and different concentrations of PLP.

We generated CARS1- and CARS2-deficient HEK293T cells and determined the intracellular levels of CysSSH derived from CARS1 and CARS2 (Fig. 9B). CysSSH was reduced to less than half in *CARS2* knockout (KO) cells, which suggests that CARS2 is the major enzyme that produces measurable persulfide/polysulfide species.

When WT CARS2 was added back to *CARS2* KO cells, however, persulfide levels increased markedly. Also, the CARS2 C78/257D mutant restored persulfide production in *CARS2* KO cells, but the K124/127A and K317/320A mutants (mutants of the KIIK and KMSK motifs, respectively) did not restore persulfide production (Fig. 9C). Thus, CARS2 acts as a CPERS in mammals, and this activity is distinct from the Cys-tRNA synthesis activity of CARS.

We investigated the CPERS activity of CARS2 *in vivo* by generating *Cars2*^+/−^ mice *via* the CRISPR/Cas9 system (Akaike et al., 2017). Although the *Cars2*^−/−^ deletion was embryonic lethal, heterozygous *Cars2*^+/−^ strain mice were born, and we observed no abnormalities in macroscopic appearance or growth profile in 6-month-old mice.

**FIG. 10.**
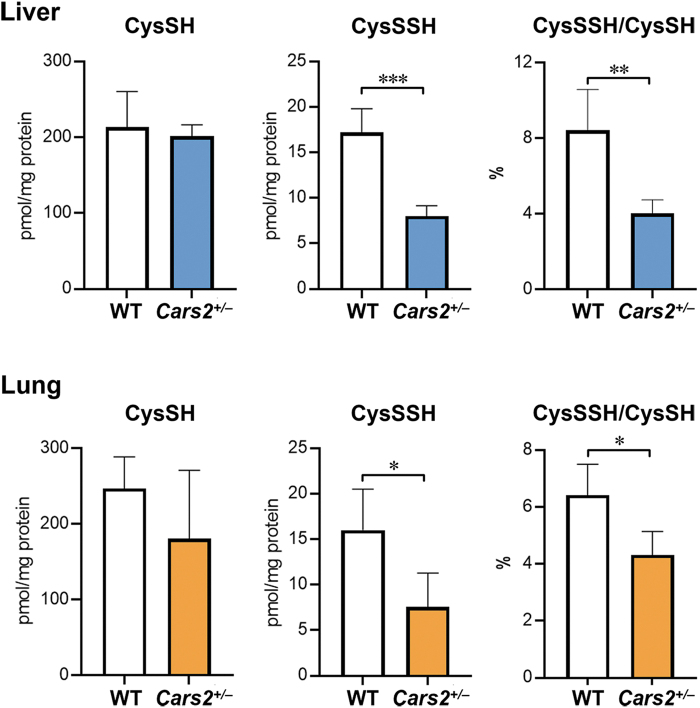
***In vivo* formation of sulfide species in WT and *Cars2*^+/−^ mice.** Endogenous production of cysteine and CysSSH was identified by means of HPE-IAM labeling LC-MS/MS analysis in livers and lungs obtained from WT and *Cars2*^+/−^ littermates (21-week-old males). The data are means ± SD (*n* = 3). *p < 0.05; **p < 0.01; ***p < 0.001.

These mice expressed only half of the mitochondrial CARS2 protein. Because the mitochondrially encoded cytochrome *c* oxidase I (MTCO1), which is a marker of mitochondrial translational function, was not substantially altered, *Cars2*^+/−^ mice probably retained Cys-tRNA synthetase activity. Sulfur metabolites in the liver and lung of *Cars2*^+/−^ mice and WT littermates were quantified by using LC-MS/MS with HPE-IAM as described earlier. Compared with WT littermates, *Cars2*^+/−^ mice had ∼50% less endogenous levels of CysSSH (Fig. 10).

To determine whether CARS2 produces and supplies CysSSH to cells, mitochondria were isolated from a mouse liver, and the amount of *de novo* synthesized CysSSH released from mitochondria was measured. In fact, mitochondria released large amounts of CysSSH. Thus, CysSSH produced in mitochondria was conceivably transferred to the cytoplasm, which led to protein polysulfidaion.

**FIG. 11.**
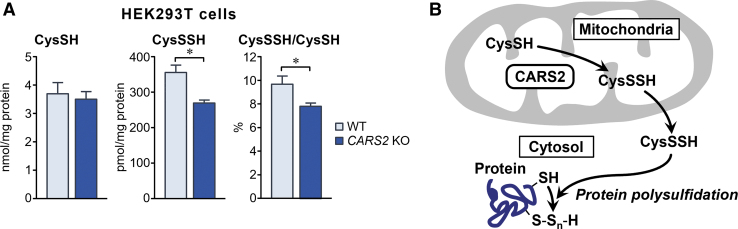
**Endogenous protein polysulfidation in HEK293T cells. (A)** The amounts of CysSSH formed in whole-cell protein recovered from WT and *CARS2* KO HEK293T cells were quantified by using HPE-IAM labeling LC-MS/MS analysis. Data are means ± SD (*n* = 3). *p < 0.05. **(B)** Schematic drawing of the mechanism of the extramitochondrial release of CysSSH into the cytosol, which may regulate whole-cell protein polysulfidation.

Indeed, the production of 20%–30% of CysSSH in whole-cell proteins (polysulfidation) depended on CARS2 expression, as identified by our *in vitro* study of cells in culture (Fig. 11A). These findings suggest that CysSSH generated from CARS2 contributed significantly to polysulfidation, mediated by post-translational and co-translational processes, and that CARS2 was a major mammalian CPERS (Fig. 11B).

## Physiological Functions of Persulfides/Polysulfides

### Regulation of redox and electrophilic signaling

**FIG. 12.**
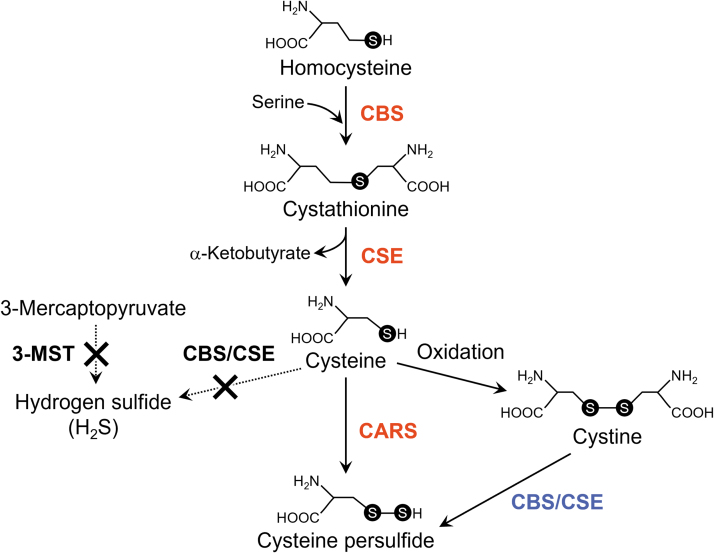
**Canonical and true pathways for persulfide biosynthesis.** The canonical pathway for persulfide production consists of sulfotransferase enzymes (CBS and CSE; *blue letters*). CARS mediates the major, true pathway that governs persulfide biosynthesis (*red letters*). 3-MST and CBS/CSE may not be involved solely in sulfide production (*black letters*). 3-MST, 3-mercaptopyruvate sulfurtransferase; CBS, cystathionine β-synthase; CSE, cystathionine γ-lyase.

In addition to CARS, cystathionine β-synthase (CBS) and cystathionine γ-lyase (CSE) generated CysSSH from cystine (Fig. 12) (Ida et al., 2014; Zainol Abidin et al., 2023). As noted previously, an endogenous sulfur transfer system that involved CysSSH produced glutathione persulfide (GSSH) at concentrations of about 10–100 μ*M* in cells in culture and *in vivo* in mammalian cells (rodents and humans).

Because reactive persulfide species including CysSSH and GSSH have higher nucleophilicity than do parental cysteine and glutathione, these reactive species demonstrated strong scavenging activities against oxidants, for example, hydrogen peroxide, and electrophiles, which contributed to the regulation of redox signaling (Ida et al., 2014).

**FIG. 13.**
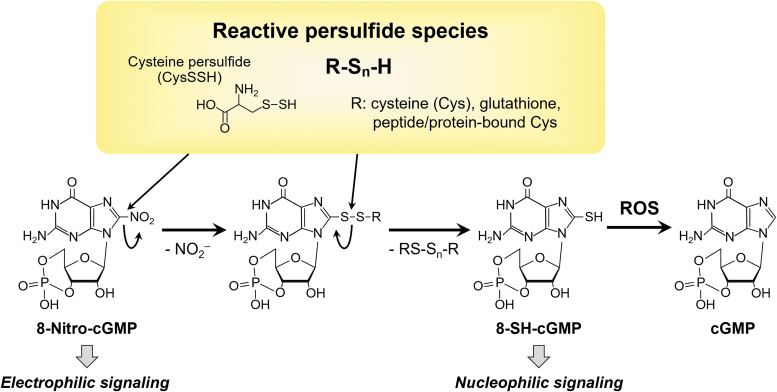
**Endogenous formation of reactive persulfide species and the function of these species in 8-nitro-cGMP metabolism.** The reactive persulfide species-dependent metabolic pathway regulates 8-nitro-cGMP signaling. 8-Nitro-cGMP reacts with reactive persulfide species to form 8-SH-cGMP, with the release of nitrite. An additional reaction between the sulfhydrated metabolite 8-SH-cGMP and ROS forms cGMP by oxidative desulfidation, and this cGMP is then degraded by phosphodiesterase. cGMP, guanosine 3′,5′-cyclic monophosphate; 8-nitro-cGMP, 8-nitroguanosine 3′,5′-cyclic monophosphate; NO_2_^−^, nitrite anion; ROS, reactive oxygen species.

Clarification of the redox signaling regulatory mechanism of reactive persulfide species including thiol-containing proteins and small thiol molecules should result in new therapeutic strategies and drug discoveries for both oxidative and electrophilic stress-related diseases. In fact, we assumed that persulfides were probably particularly nucleophilic and reducing, which was the case, because they reacted quickly with hydrogen peroxide and a biologically generated electrophile 8-nitroguanosine 3′,5′-cyclic monophosphate (Fig. 13).

These data indicate that persulfides may be important signaling and effector species, and because H_2_S can be a product of persulfide degradation, much of the reported biological activity associated with H_2_S may actually be persulfide activity. That is, H_2_S may act mainly as a marker of biologically active persulfide species.

### Polysulfidation-mediated regulation of oxidative stress in proteins

Another possible protein polysulfidation function, in addition to regulating redox signaling, is protecting protein thiol residues against irreversible chemical modification that oxidants and electrophiles cause. Living organisms rely strongly on redox chemistry, which transfers electrons among various molecules to synthesize and metabolize molecules and maintain vital activity.

Although cysteine residues in proteins are critically involved in this redox reaction, when a cysteine thiol (R-SH) is irreversibly and excessively oxidized to sulfinic acid (R-SO_2_H) and sulfonic acid (R-SO_3_H), functions of oxidized proteins are significantly reduced. CysS-S_n_-H formed on proteins are oxidized more easily than are their parental cysteine residues.

**FIG. 14.**
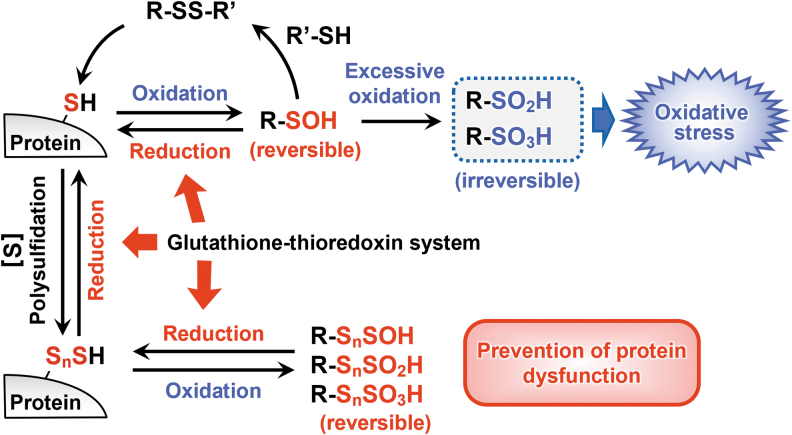
**Mechanisms to avoid oxidative damage to proteins caused by persulfides.** A protein thiol (Protein-SH) is irreversibly oxidized by being excessively oxidized, but persulfides have a reducing ability and can provide a functionally reversible oxidation state, protein persulfidation (Protein-S_n_H). The glutathione-thioredoxin system contributes to the reduction of excessively oxidized persulfides, perthiosulfinic acid (R-S_n_SO_2_H), and perthiosulfonic acid (R-S_n_SO_3_H).

The stepwise oxidation of a persulfide group leads to consecutive formation of perthiosulfenic acid (R-SSOH), perthiosulfinic acid (R-SSO_2_H), and perthiosulfonic acid (R-SSO_3_H) (Ono et al., 2014). A recent report demonstrated a mechanism by which cells protect proteins from irreparable damage caused by reactive oxygen species (Dóka et al., 2020). Unlike R-SO_2_H and R-SO_3_H, which are irreversibly oxidized forms, a hydrosulfide group (-SH) can be regenerated from R-SSO_2_H and R-SSO_3_H by reductive cleavage of the disulfide bond, which is interpreted as escaping from oxidative damage and preventing functional deterioration of the protein (Fig. 14).

That is, persulfides protect proteins from irreversible modification by electrophiles (Takata et al., 2019). The glutathione-thioredoxin system, which is the main cellular reducing system, usually reduces and repairs oxidized cysteines. Investigation of protein cysteine residues in mice that had a defective glutathione-thioredoxin system showed that they had reduced cysteine and increased R-SSO_3_H compared with WT mice.

This finding indicates that R-SSO_2_H and R-SSO_3_H are present in abundance in mice and protect proteins by preventing their oxidation. Protein polysulfidation, thus, plays an extremely important role in living organisms. We demonstrated that the novel mechanism not only protects proteins but also regulates and modifies their functions. Phosphatase and tensin homolog deleted from chromosome 10, protein-tyrosine phosphatase 1B, peroxiredoxins, and heat shock protein 90 are good examples of these functions (Dóka et al., 2020).

These pathways are generally thought to decrease under oxidative conditions, including aging and aging-related diseases. In this context, the antioxidant function of persulfides seems to contribute to the antibiotic resistance of various bacteria (Ono et al., 2021; Shatalin et al., 2011).

### Regulation of ferroptosis in eukaryotes

Ferroptosis is a unique mode of cell death driven by iron-catalyzed free radical oxidation reactions, which result in damage or modification of biological membranes by means of lipid peroxidation (Jiang et al., 2021). The pharmacological modulation of ferroptosis, *via* its induction and inhibition, holds great potential for treating drug-resistant cancers, ischemic organ injuries, and other degenerative diseases associated with extensive lipid peroxidation (Barayeu et al., 2023).

**FIG. 15.**
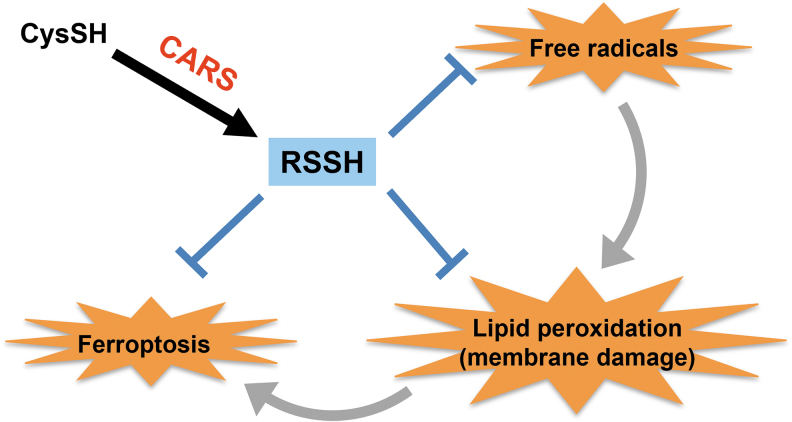
**Inhibition by CARS-generated persulfides/polysulfides of peroxidation and ferroptosis by scavenging radicals.** RSSH, hydropersulfides.

In a recent study, we demonstrated that hydropolysulfides scavenged endogenous free radicals, thus suppressing lipid peroxidation and ferroptosis (Fig. 15) (Barayeu et al., 2023). They and Wu et al. (2022) showed that hydropolysulfides could act as potent inhibitors of O_2_-dependent membrane damage and destruction, which made them essential regulators and inhibitors of ferroptosis.

Fukuto (2023) also reported that if hydropolysulfides are endogenous and inducible antioxidants/reductants, they may have functions other than inhibiting ferroptosis. Thus, hydropolysulfides may be utilized to regulate ferroptosis and to develop effective drugs for treating various diseases.

### Supersulfide catalysis for redox-dependent enzymes and signal regulation

Discovery of the conserved production of persulfides/polysulfides among organisms and their diverse biological consequences led us to conceptually define biologically functioning persulfides/polysulfides as “supersulfides.” In fact, supersulfides include hydropersulfide (RSSH) species and polymeric sulfurs with sulfur catenation (RSS_n_R, *n* > 1, R = hydrogen or alkyl, or cyclized sulfurs), which are all biologically relevant sulfur compounds and allotropes, but cannot be fully covered by other terms such as reactive sulfur species (RSS), sulfane sulfurs, and persulfides/polysulfides (Matsunaga et al., 2023).

They are now widely recognized as universal bioactive metabolites formed physiologically in all organisms (Fukuto, 2018; Ida et al., 2014; Ono et al., 2014; Saund et al., 2015). For example, our earlier study indicated that activity of the persulfide dioxygenase (*e.g.,* ethylmalonic encephalopathy protein 1 [ETHE1]), which is a major enzyme that metabolizes endogenous GSSH and glutathione polysulfide GS-S_n_-H, was maintained by means of supersulfide catalysis of the Cys^247^ residue (Jung et al., 2016).

This Cys^247^ is located near the iron ion at the active center of ETHE1 (Pettinati et al., 2015). In fact, ETHE1 reportedly metabolizes GSSH to glutathione with simultaneous oxygen consumption. How ETHE1 activity is regulated is still unclear, however. We first found that ETHE1 catalyzed the persulfide dioxygenase reaction mostly for GS-S_n_-H and GSSH but not for other endogenous persulfides such as cysteine and homocysteine persulfides/polysulfides.

PMSA analysis then indicated that most cysteine residues in ETHE1 were polysulfidated. Site-directed mutagenesis of cysteine residues in ETHE1 combined with LC-MS/MS for polysulfidation determination surprisingly indicated that the Cys^247^ residue was the site most effective for supersulfide formation, that is, polysulfidation of the cysteine residue, and that the C247S mutant possessed no persulfide dioxygenase activity.

These results suggested that ETHE1 is a major enzyme regulating endogenous GSSH/GS-S_n_-H and that its activity is controlled by supersulfide catalysis at the active site of the Cys^247^ residue. We also found recently that supersulfides catalyzed nitric oxide (NO) metabolism *via* a glutathione-dependent electron transfer from aldehydes by exploiting ADH5 (Kasamatsu et al., 2023).

ADH5 is a highly conserved bifunctional enzyme serving as *S*-nitrosoglutathione reductase (GSNOR) that downregulates NO signaling and formaldehyde dehydrogenase that detoxifies formaldehyde in the form glutathione hemithioacetal. The C174S mutation significantly reduced ADH5 polysulfidation and almost abolished GSNOR activity but spared formaldehyde dehydrogenase activity. *Adh5^C174S/C174S^* mice showed improved cardiac functions possibly because of GSNOR elimination and consequently increased NO bioavailability.

Supersulfides in ADH5, thus, constitute a substantial catalytic center for NO metabolism mediating electron transfer from aldehydes. We should note that both ETHE1 and ADH5 are highly conserved among prokaryotes and eukaryotes.

### Sulfide-dependent energy production in prokaryotes

**FIG. 16.**
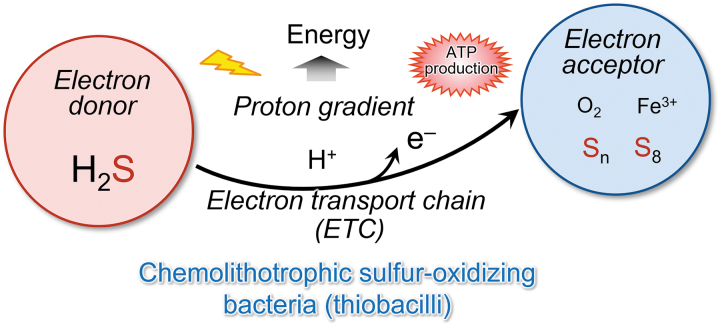
**Energy production in bacteria that utilizes sulfide.** Chemolithotrophic sulfur-oxidizing bacteria such as *Thiobacillus* utilize H_2_S as an electron donor to produce a proton gradient and produce energy *via* the ETC.

In bacteria, sulfide can be an electron donor (Aussignargues et al., 2012; Nübel et al., 2000; Shahak and Hauska, 2008). *Aquifex aeolicus*, a hyperthermophilic and microaerophilic bacterium, obtains energy for growth from only inorganic compounds. A previous proposal was that one respiratory pathway in this organism consists of an electron transfer from H_2_S to molecular oxygen (Fig. 16) (Nübel et al., 2000).

H_2_S is oxidized by the sulfide:quinone oxidoreductase (SQR), a membrane-bound flavoenzyme that reduces the quinone pool. These data constitute an experimental demonstration of the existence of such a respirasome with sulfide oxidase-oxygen reductase activity. Thus, two different bioenergetic pathways (sulfur reduction and sulfur oxidation) exist in this bacterium as supramolecular structures in the membrane (Prunetti et al., 2010).

In fact, archaea organisms reportedly utilize sulfur compounds, including persulfides, as electron donors or electron acceptors for energy production (Arnulf, 2006). Various types of dissimilatory sulfur metabolism, that is, reactions used for energy conservation, occur in archaea, both the Crenarchaeota and the Euryarchaeota phyla.

Although these reactions are not yet fully characterized, major processes include aerobic elemental sulfur (S^0^) oxidation, anaerobic S^0^ reduction, anaerobic sulfate/sulfite reduction, and anaerobic respiration of organic sulfur (Liu et al., 2012). These reports suggested that persulfides are used for energy metabolism in the domains Bacteria and Archaea.

### Mitochondrial energy metabolism in eukaryotes

In the mitochondrial inner membrane, electrons are transported from NADH to the final electron acceptor, the oxygen molecule. The proton gradient and ATP production are linked to electron transport. We showed that the mitochondrial isoform CARS2 is involved in forming and maintaining the mitochondrial membrane potential (Akaike et al., 2017).

We believe that it is quite plausible that CysSSH generated by mitochondrial CARS2 contributes to mitochondrial energy metabolism *via* formation of the membrane potential. Also, we clarified a different profile of the products of human CARS2 in the cell-free enzyme reaction compared with cellular CARS2 metabolism in HEK293T cells in culture (Akaike et al., 2017).

Although CARS2 mainly synthesized CysSSH/SSSH in a cell-free solution, preferential formation of HS^−^ and thiosulfate over CysSSH was evident with HEK293T cells. Thus, we hypothesized that the mitochondrial compartment is a unique metabolic environment in which *de novo* CysSSH synthesized by CARS2 may be further metabolized, possibly coupled with the mitochondrial electron transport chain (ETC). Also, we examined the effect of ETC suppression on the metabolic profile of CysSSH and its derivatives in HEK293T cells.

The ETC-suppressive treatments caused a significant increase in CysSSH and a simultaneous reduction of HS^−^ production. We interpret these results to mean that CysSSH and/or related polysulfide metabolites derived from CARS2 in mitochondria could be reduced by accepting an electron from the ETC to release HS^−^ (H_2_S). It is, thus, suggested that CysSSH acts as an electron acceptor that is reduced to H_2_S in an electron transport-dependent manner, and this H_2_S acts as an electron donor for the ETC (Akaike et al., 2017).

**FIG. 17.**
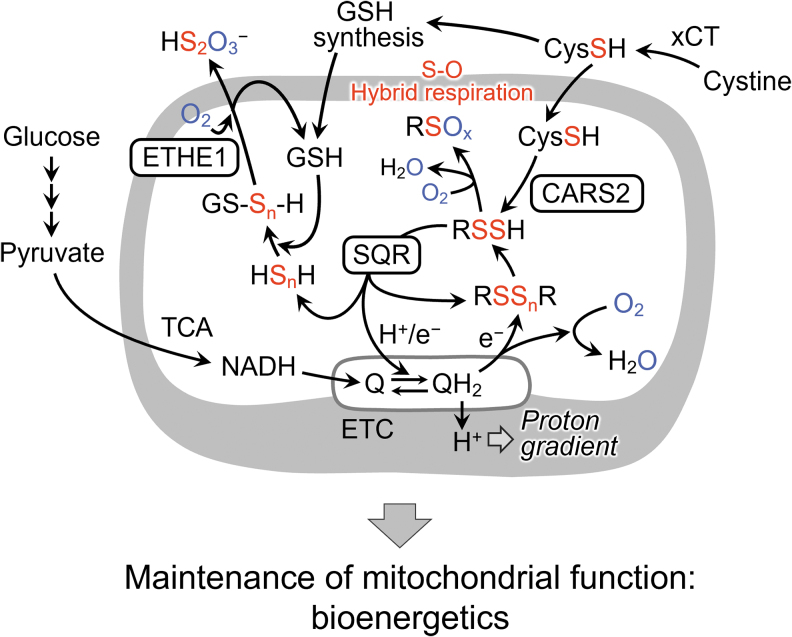
**Mitochondrial sulfur respiration: Energy production by means of electron transfer conjugation of CARS2-derived persulfides.** CysS-S_n_-H produced by CARS2 in mitochondria may be reductively metabolized to sulfides and then oxidized by the SQR, in a manner linked to the ETC in mitochondria. CysS-S_n_-H-dependent sulfur metabolism is coupled with formation of glutathione polysulfide (GS-S_n_-H), which is controlled by the mitochondrial ETC. CARS2, mitochondrial cysteinyl-tRNA synthetase; ETHE1, ethylmalonic encephalopathy protein 1 (persulfide dioxygenase); GS-S_n_-H, glutathione hydropersulfide/polysulfide; Q/QH_2_, ubiquinone/ubiquinol; SQR, sulfide:quinone oxidoreductase; TCA, tricarboxylic acid; xCT, cystine/glutamic acid transporter.

These data demonstrate the discovery of sulfur respiration in the biological world and the rediscovery of sulfur respiration in humans and other mammals. In this newly discovered sulfur respiration, the final electron acceptors in the electron transfer system are persulfides. In oxygen respiration, electrons from ETCs are transferred to oxygen to produce water, but in sulfur respiration, electrons are transferred to CysSSH or various polysulfides to produce H_2_S (Fig. 17).

In fact, SQR proteins localize in mitochondria and provide protons and electrons derived from sulfide and hydropersulfide to the mitochondrial ubiquinone (Q: mammalian coenzyme Q10) cycle. A recent report demonstrated that SQR-mediated oxidation of sulfide promoted reverse electron transport in mitochondrial complex I (Jia et al., 2020).

Thus, CysSSH produced by CARS2 in mitochondria may be reductively metabolized to cysteine and HS^−^, which are then oxidized by SQR and linked to the mitochondrial ETC. Also, we have recently revealed that NRF2 is involved as part of the activation mechanism of SQR-mediated sulfur respiration in mitochondria (Alam et al., 2023). Further studies will reveal more detailed mechanisms. The discovery of sulfur respiration in mammals will have a significant impact not only on basic biology but also on the pathogenesis of diseases related to energy metabolism.

Our previous study also demonstrated novel physiological roles of CARS as a CPERS during the regulation of mitochondrial functions (Akaike et al., 2017). *CARS2* KO cells manifested markedly modified mitochondrial morphology (*i*.*e*., a shrunken or fragmented appearance), which greatly improved after we added back CARS2, as indicated by MitoTracker Red fluorescence staining, transmission electron microscopy, and immunofluorescence staining of translocase of outer mitochondrial membrane 20 and CARS2.

Unlike the other Lys mutants that we tested, the C78/257D mutant and the WT CARS2 showed much improved mitochondrial morphology. Relevant to these results, deletion of CARS2-activated dynamin-related protein 1 (Drp1), which is a primary mitochondrial fission mediator (Akhtar et al., 2016), and adding back WT CARS2 and the C78/257D mutant but not the K317/320A mutant significantly reduced Drp1 GTPase activity, thereby producing CysSSH without CARS activity.

During normal culture environments, Drp1 in HEK293T cells was extensively polysulfidated, as our new biotin-PEG-MAL capture method demonstrated. Both *CARS2* KO and *CARS1/2* double-knockdown cells had greatly reduced Drp1 polysulfidation. Drp1 was likely activated by chemical depolysulfidation or by a post-translational process that was sustained physiologically by the thioredoxin-thioredoxin reductase system, for example. We, thus, determined that Drp1 is an important signal effector molecule that is reversibly regulated *via* a unique process involving polysulfidation and depolysulfidation.

Data clearly showed that CARS2 contributed to mitochondrial biogenesis and functioning. In particular, mitochondrial DNA that was normalized by nuclear DNA was reduced in *CARS2* KO cells but recovered after WT CARS2 and the C78/257D mutant, but not the Lys mutants, were added back, which suggested that CARS2-derived persulfide enhanced mitochondrial biogenesis.

In *CARS2* KO cells, the mitochondrial membrane potential decreased, but it recovered or increased after the WT and the C78/257D mutant were added back or overexpressed but not after using the Lys mutants. We used extracellular flux analysis to measure the oxygen consumption rate (OCR) in HEK293T *CARS2* KO cells. The OCR in these cells was about 50% of that in WT cells, a finding that agrees with the partial elimination of the CARS2 protein and thus a decreased MTCO1 expression in *CARS2* KO cells.

Adding the WT CARS2 and the C78/257D mutant, but not the Lys mutant, improved the reduced OCR in *CARS2* KO cells. These data led us to suggest the new concept that CARS2-derived CysSSH plays a substantial role in the mitochondrial ETC, which allowed us to theorize a completely new and fundamental role of persulfides in maintaining mitochondrial bioenergetics.

As we investigated how CARS2-derived CysSSH supports mitochondrial bioenergetics, we observed a truly different profile of human CARS2 products in a cell-free enzyme reaction compared with cellular CARS2 metabolism in cultured HEK293T cells. CARS2 synthesized mostly CysSSH/CysSSSH in cell-free solutions, but in HEK293T cells, the hydrosulfide ion HS^−^ (from H_2_S) together with thiosulfate, rather than CysSSH, preferentially formed.

We, thus, postulated that the mitochondrial compartment is a unique metabolic environment for additional metabolism of *de novo* CysSSH synthesized by CARS2, possibly coupled with the mitochondrial ETC.

Our study of the metabolic profile of CysSSH and its derivatives in HEK293T cells found that CARS2-dependent CysSSH production and ETC function had a close relationship. We used two methods to inhibit cellular ETC and thus bring about a loss of mitochondrial DNA. In one method, we used the specific inhibitor of complex III antimycin A; in the other method, we used the ETC disrupter ethidium bromide.

Both treatments led to significantly increased production of CysSSH and a simultaneous reduction in HS^−^ production, as evidenced by using HPE-IAM labeling LC-MS/MS analysis. These opposite, stoichiometric relationships between CysSSH and H_2_S formation led us to believe that the conversion of CysSSH to H_2_S depended on ETC activity and was mediated by cellular ETC. Thus, CysSSH generated from CARS2 in mitochondria is conceivably reduced by accepting an electron from the ETC, thus producing H_2_S.

These data also give strong support for the CARS2-CysSSH pathway involvement in mitochondrial functioning, because the production of CARS2-dependent CysSSH is integrated into and tightly linked to the mitochondrial ETC, which is itself involved in energy metabolism. Studies have reported that low H_2_S concentrations (in the nanomolar range) maintained the ETC function that may have been mediated by SQR and additional enzymes that oxidize sulfides to thiosulfate (Goubern et al., 2007; Griesbeck et al., 2002; Grieshaber and Völkel, 1998; Hine et al., 2015; Ono et al., 2014; Szabo et al., 2014).

However, the way in which endogenous H_2_S was supplied in mitochondria remained unclear. Our studies suggested that CSE, CBS, and 3-mercaptopyruvate sulfurtransferase are not the main H_2_S sources in mitochondria in various mammalian cell lines and *in vivo* in mice (Mishanina et al., 2015; Morikawa et al., 2012; Nakano et al., 2015; Nishida et al., 2012; Ono et al., 2014; Shirozu et al., 2014; Yadav et al., 2016). Indeed, one of our studies was the first to confirm the indirect formation of HS^−^ (or H_2_S) from CARS2 *via* CysSSH generation in mitochondria (Akaike et al., 2017).

Also, another study determined that CysSSH may be partly responsible for endogenous iron-sulfur cluster formation (Takahashi et al., 2017). Because these clusters are synthesized and used in complexes I–III of the ETC in mitochondria (Stehling and Lill, 2013) and are actively transported to outside the mitochondria, CysSSH-dependent HS^−^ metabolism may be associated with the production of iron-sulfur centers of the mitochondrial ETC and with the cytosolic formation and maintenance of different iron-sulfur complex systems. CARS2, therefore, acts as a major CPERS that benefits mitochondrial biogenesis and bioenergetics.

### Persulfide function in hypoxia

The mammalian brain is highly sensitive to a lack of oxygen, but the mechanism determining the brain's susceptibility to hypoxia is not completely understood. We determined that the sensitivity of the brains of mice, rats, and naturally hypoxia-tolerant ground squirrels to hypoxia is inversely related to the SQR levels and the ability to catabolize sulfide.

Silencing SQR led to an increase in the brain's sensitivity to hypoxia, whereas neuron-specific SQR expression blocked hypoxia-induced sulfide accumulation, bioenergetic failure, and ischemic brain injury (Marutani et al., 2021). Removing SQR from mitochondria caused an increase in sensitivity to hypoxia not only in the brain but also in the heart and liver.

Pharmacological scavenging of sulfide, hydroxocobalamin, sustained mitochondrial respiration in hypoxic neurons so that mice were resistant to hypoxia. These findings illustrate the essential role of sulfide catabolism in energy homeostasis during hypoxia and point to a therapeutic target in ischemic brain injury (Marutani et al., 2021).

### Anti-inflammatory effects of polysulfides in eukaryotes

We reported in an earlier study that the increase in cellular polysulfides by using polysulfide donors, *N*-acetyl-l-cysteine (NAC) polysulfide, that we developed caused considerable inhibition of lipopolysaccharide-initiated macrophage activation (Zhang et al., 2019). We have revealed that NAC polysulfide can donate their sulfur atoms to acceptor thiols such as GSH, which would result in the production of hydropersulfides/polysulfides (such as CysSSH, GSSH, GSSSG) *in vitro* and in cells.

Treatment with polysulfide donors caused a strong suppression of lipopolysaccharide-induced pro-inflammatory responses in macrophages by inhibition of Toll-like receptor 4 (TLR4) signaling. Polysulfide donor treatment also significantly reduced pro-inflammatory responses induced by other TLR signaling stimulants. Administration of polysulfide donors protected mice from mortality induced by lethal endotoxin shock. These findings show that cellular polysulfides negatively regulate TLR4-mediated pro-inflammatory signaling, thus becoming a possible target for inflammatory disorders.

## Concluding Remarks

**FIG. 18.**
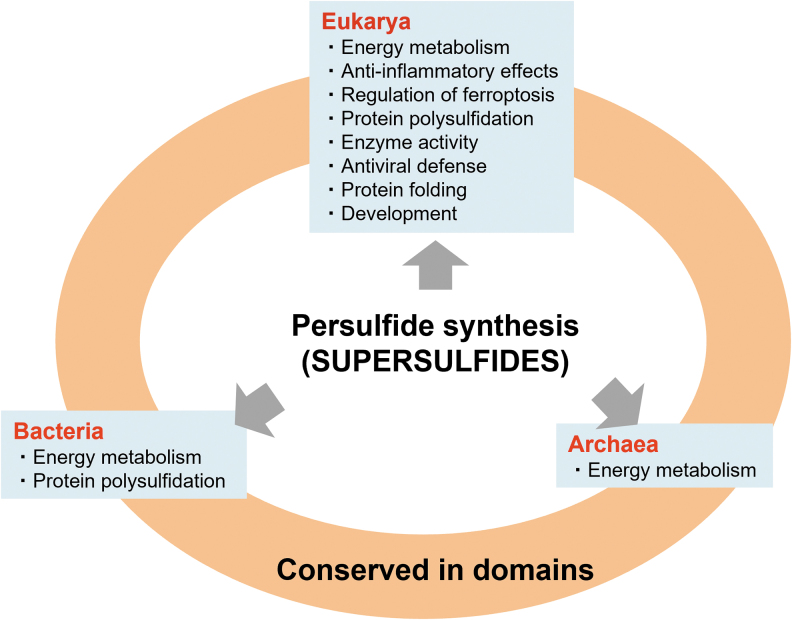
**Evolutionarily conserved persulfide biosynthesis in organisms.** Persulfide synthesis regulates energy metabolism in the domain Bacteria, Archaea, and Eukarya. In eukaryotes, persulfides have various physiological functions, including anti-inflammatory effects, regulation of ferroptosis, and antiviral defense.

In summary, this review described recent advances in persulfide/polysulfide research, including the development of analytical technology for persulfide/polysulfide quantification and for clarifying the mechanisms of persulfide biosynthesis in the domains Bacteria, Archaea, and Eukarya (Fig. 18). The supersulfides, which conceptually represent the diverse and versatile persulfides/polysulfides that are biologically produced in all organisms, are closely related to almost all life-sustaining phenomena, as thoroughly discussed in this review.

Recently, genome-wide association studies revealed that the reduction of CARS2 expression is associated with a specific disease risk (Dang et al., 2022). This study indicated that persulfide formation is essential for preventing diseases. Therefore, the recent discovery of the biological formation of diverse polymeric sulfurs or sulfur allotropes now warrants elucidation so as to understand the physiological functions of persulfides/polysulfides or supersulfides.

This new concept of supersulfides may facilitate the development of various preventive and therapeutic measures for different diseases that are caused by oxidative stress and redox-based pathobiology.

## Abbreviations Used

3-MST: 3-mercaptopyruvate sulfurtransferase
8NcG: 8-nitro-cGMP
8-nitro-cGMP: 8-nitroguanosine 3′,5′-cyclic monophosphate
ADH5: alcohol dehydrogenase 5
ASBT: 2-aminosulfonyl benzothiazole
biotin-PEG-MAL: biotin-polyethylene glycol-maleimide
BPM: biotin-PEG-MAL
CARS: cysteinyl-tRNA synthetase
CBB: Coomassie Brilliant Blue
CBS: cystathionine β-synthase
cGMP: guanosine 3′,5′-cyclic monophosphate
CPERS: cysteine persulfide synthase
CSE: cystathionine γ-lyase
CysSH: cysteine
CysSSH: cysteine persulfide
CysS-S_n_-H: cysteine hydropersulfide/polysulfide
Cys-tRNA: cysteinyl-tRNA
Drp1: dynamin-related protein 1
DTNB: 5,5′-dithiobis(2-nitrobenzoic acid)
DTP: 4,4′-dithiopyridine
EcCARS: CARS from *Escherichia coli*
ESI: electrospray ionization
ETC: electron transport chain
ETHE1: ethylmalonic encephalopathy protein 1
GAPDH: glyceraldehyde-3-phosphate dehydrogenase
GS-S_n_-H: glutathione hydropersulfide/polysulfide
GSNOR: *S*-nitrosoglutathione reductase
GSSH: glutathione persulfide
H_2_S: hydrogen sulfide
Hap: heme activator protein
HPE-IAM: β-(4-hydroxyphenyl)ethyl iodoacetamide
KO: knockout
LC-MS/MS: liquid chromatography-tandem mass spectrometry
LMW: low-molecular-weight
Lys: lysine
MBB: monobromobimane
MMTS: methyl methanethiosulfonate
MSBT: 2-methylsulfonyl benzothiazole
MTCO1: mitochondrially encoded cytochrome *c* oxidase I
NAC: *N*-acetyl-l-cysteine
NEM: *N*-ethylmaleimide
NO: nitric oxide
NO_2_^−^: nitrite anion
OCR: oxygen consumption rate
PCMB: *p*-chloromercuribenzoic acid
PLP: pyridoxal phosphate
PMSA: PEG-conjugated maleimide labeled gel shift assay
PUNCH-P: puromycin-associated nascent chain proteomics
PUNCH-PsP: PUNCH-P modified for polysulfide proteomics
Q-TOF: quadrupole time-of-flight
ROS: reactive oxygen species
R-S_n_-H: hydropolysulfides
R-SSO_2_H: perthiosulfinic acid
R-SSO_3_H: perthiosulfonic acid
SD: standard deviation
Sep: *O*-phosphoserine
SepCysS: Sep-tRNA:Cys-tRNA synthase
siRNA: small interfering RNA
SQR: sulfide:quinone oxidoreductase
TLR4: Toll-like receptor 4
WT: wild type
